# Highlight report: The relationship of DNA copy number alterations and mRNA levels in cancer

**DOI:** 10.17179/excli2017-1043

**Published:** 2017-12-21

**Authors:** Seddik Hammad

**Affiliations:** 1 Department of Forensic Medicine and Veterinary Toxicology, Faculty of Veterinary Medicine, South Valley University, 83523-Qena, Egypt

## ⁯⁯⁯

Recently, a genome-wide study about gene copy number gains and corresponding expression levels has been analyzed in a cohort of 190 non-small cell lung cancer (NSCLC) patients (Jabs et al., 2017[5]). The authors report that approximately half of the analyzed gene copy number-gene expression pairs correlated significantly. However, only 1.6 % (corresponding to 301 genes) of the analyzed pairs (gene copy number- mRNA) showed very strong correlations with a correlation coefficient higher than 0.7 (Jabs et al., 2017[5]). The authors studied the location of these 301 genes and observed that they are found predominantly in 10 chromosomal `hotspot regions´ with a width of approximately 15 Mbp. The most probable explanation of these `hotspots´ is that these genes show higher mean expression levels; moreover, copy number variations are more likely in these regions. Both, mRNA expression level and the frequency of copy number gain in the patient cohort were significantly associated with the correlation coefficient of copy number-gene expression pairs. An interesting result is that some of the genes with high correlation between copy number and mRNA levels are significantly associated with survival. Indeed, prognostic genes were overrepresented among the subset of highly correlating copy number-gene expression pairs (Jabs et al., 2017[5]). 

Since decades, much effort has been invested to better understand the relationship between gene expression and prognosis of tumors (Hellwig et al., 2016[4]; Stock et al., 2015[13]; Sicking et al., 2014[12][11]; Ghallab et al., 2015[2]; Malik et al., 2015[9]; Lohr et al., 2015[8]; Shakeri et al., 2016[10]). However, it has also become clear that it is challenging to achieve a relevant improvement of prognostication by gene expression signatures compared to the use of clinicopathological parameters alone (Grinberg et al., 2017[3]; Sicking et al., 2014[12][11]). A further research focus is the identification of improved anti-cancer agents and the identification of subsets of patients who profit from a specific chemotherapy (Uzor, 2016[15]; Jigyasu et al., 2016[6]; Kwak et al., 2016[7]; Benarba, 2015[1]; Tatokoro et al., 2015[14]). It is often easier to isolate DNA from archived tumor material than obtaining the easily degradable mRNA. The systematic study of Jabs et al. identified a subset of genes with a very strong correlation of DNA copy number and mRNA levels. Therefore, further analysis of prognostic or predictive relevance of these genes can be performed based on DNA which may facilitate progress in this field of research. 
